# The impact of leucine supplementation on body composition and glucose tolerance following energy restriction: an 8-week RCT in adults at risk of the metabolic syndrome

**DOI:** 10.1038/s41430-023-01360-1

**Published:** 2023-11-03

**Authors:** Kaveri Pathak, Yun Zhao, Emily K. Calton, Anthony P. James, Philip Newsholme, Jill Sherriff, Mario J. Soares

**Affiliations:** 1 Curtin School of Population Health, Curtin University, Bentley Campus, Perth, WA 6102 Australia; 2 Curtin Medical School, Curtin University, Bentley Campus, Perth, WA 6102 Australia

**Keywords:** Biomarkers, Metabolism

## Abstract

**Background:**

L-Leucine (Leu) supplementation may benefit fat-free mass (FFM) per se and glucose metabolism.

**Objectives:**

To determine whether Leu supplementation during energy restriction blunted the loss of FFM, enhanced the loss of fat mass (FM) and improved glucose tolerance.

**Design:**

Thirty-seven adults, aged 20–65 years, with increased waist circumference and at least one other metabolic syndrome (MetS) component, were selected. We employed a two-arm parallel, double blind, randomized control trial (RCT) design. Participants were randomly assigned to an intervention group (leucine – 3 g/d) or placebo (lactose - 2.67 g/d), while following an individualised energy restricted diet for an 8-week period. Detailed body composition (DEXA), oral glucose tolerance test (OGTT), insulin and components of MetS were measured before and after the trial. Analysis of covariance (ANCOVA) assessed the effect of Leu on an intention-to-treat (ITT) principle. Bootstrapping method with 1000 bootstrap samples was used to derive parameter estimates, standard errors, *p*-values, and 95% confidence intervals for all outcomes.

**Results:**

Adjusted for baseline values and other covariates, FFM (*p* = 0.045) and lean tissue mass (LTM) (*p* = 0.050) were significantly higher following Leu. These outcomes were modified by a significant treatment x sex interaction that indicated Leu had the greater effect in men. However, on adjustment for body composition changes, there was no difference in insulin sensitivity, oral glucose tolerance, or change in MetS components following Leu.

**Conclusion:**

Short-term leucine supplementation during energy restriction resulted in a greater preservation of FFM and LTM particularly in men, but did not impact glucose metabolism.

## Introduction

Excess body weight is a leading factor in the etio-pathogenesis for most non-communicable chronic diseases (NCDs) that includes cardiovascular disease, type 2 diabetes, stroke and cancer. Excess weight also accounts for the associated greater rates of morbidity, disabilities and mortality worldwide [1]. Obesity and NCD combined are an escalating challenge to public health, despite progress in primary prevention and in medical therapy. In Australia, deaths due to cancer and cardiovascular diseases have more than doubled from 23% in 1915 to 58% in 2015 [2], and NCDs accounted for 71% of global deaths [3]. The National Health and Nutrition Examination Survey (NHANES) has clearly established a positive association between increased body weight and prevalence of metabolic syndrome (MetS) (22% in overweight and 60% in obese) [4]. The risk of MetS doubles with advancing age (10% in those aged 20–29; 20% 40–49 & 45% 60–69) [5] and the, rates of mortality and morbidity will vary with the number of components present within each individual [6]. Weight loss is one of the best approaches in reducing the burden of NCDs [7]. Loss of FFM during weight loss is a common occurrence but the proportions may vary during surgical or non-surgical (diet, exercise, medications) weight-loss interventions. Moreover, the proportion of FFM lost depends on the age, gender and the degree of energy restriction, protein intake, exercise, pharmaceutical and surgical intervention [8]. This is critical since FFM is strong predictor of resting metabolic rates (RMR) and a decreased RMR may increase risk of weight gain [9]. Additionally, a reduced FFM is a strong independent predictor of increased insulin resistance, which in turn increases the risk for several chronic diseases [10].

Leucine is one of the three amino acids involved in stimulating protein synthesis and activation of mRNA via activation of nutrient signalling [11]. Activation of the mechanistic target of rapamycin complex 1 (mTORC1) by leucine has demonstrated its ability to enhance the muscle protein synthetic machinery independently, specifically playing the role in the translation initiation process [12, 13]. One-third of our minimum daily requirement for essential amino acids is met by the three branched chain amino acids (BCAA): leucine, isoleucine and valine [14]. An expert group have projected that the leucine requirements may be up to four times greater than that suggested in the landmark 1985 WHO/FAO/UNU report [15]. High protein diets preserve muscle mass during weight loss [16], and consumption of whey protein is particularly effective for increasing muscle protein synthesis [17]. Leucine is found in relatively high quantities in whey protein (approx. 10%) and accelerates protein anabolism, cell growth and metabolism [12, 18] where leucine acts as a substrate but also a “trigger” in the process of protein synthesis [19]. It is postulated that anabolic competence of ingested amino acids in muscle protein synthesis is characterised by essential amino acids profiles, bioavailability and mechanisms by which they are delivered to muscle tissues [20].

Leucine’s capacity to influence insulin secretion [21] and improve insulin sensitivity and reduce cardiovascular risks [22] has been documented. Several studies on leucine supplementation indicate an increase in muscle mass [23] especially appendicular muscle mass [24], increased resting energy expenditure and lower respiratory quotient (RQ) indicating greater fat oxidation [25, 26]. Leucine may also increase satiety with an additional impact on glucose metabolism [27, 28] and stimulate energy and lipid metabolism by regulating uncoupling protein-3 (UCP-3) expression in metabolically active tissues such as skeletal muscle, brown adipose tissue (BAT) and white adipose tissue (WAT) [25, 29]. The primary objective of this trial was to explore if short-term leucine supplementation during a energy-restricted diet would enhance fat loss and preserve FFM and/or lean tissue mass (LTM) in centrally obese adults at risk of MetS.

## Methods

### Participant selection

MetS is a cluster of conditions that occur together and increase the risk of chronic diseases such as heart disease, stroke and type 2 diabetes. To be diagnosed with MetS, a person must have at least 3 of the following criteria: Abdominal obesity (80 cm or more in women and 84 cm or more in men), raised blood pressure (130/85 mmHg or higher), raised blood sugar levels (100 g/dL or higher), raised triglyceride levels (150 mg/dL or higher) or low HDL cholesterol levels (less than 40 mg/dL in men & 50 mg/dL in women). Thirty seven obese adult men and women of European origin (20–65 years) with abdominal obesity as assessed by waist circumference and at least one additional criterion for MetS were recruited for this study [30]. We excluded those with a history of myocardial infarction (MI), stroke, type 1 diabetes, polycystic ovarian syndrome and thyroid disease. Potential participants who reported intentional weight loss in last 6 months, were pregnant or lactating, on any medication that was likely to affect body composition, energy expenditure or food intake or on hormonal contraceptives or replacement therapy were also excluded. The study included one participant with type 2 diabetes who had good glucose control (HbA1C < 6.5%). Those on calcium or vitamin D supplements ceased intake two weeks prior to first measurement day. Other medications for lowering lipids, glucose and blood pressure were noted, and their dose monitored throughout the study period for any change.

### Study design, ethics and randomisation

This was two-arm parallel, double-blind RCT comparing leucine and placebo supplementation during an eight-week energy restricted dietary program. Random allocation software [31] was used to allocate participants to either experimental [leucine-3 g/d] and control groups [lactose 2.67 g/d]. Treatment codes were revealed only on study completion. The investigator (KP) who carried out the trial, other researchers involved, and all participants were unaware of the treatment allocation since the codes were held by JS.

The human research ethics committee (HREC) of Curtin University provided ethical approved the study (HREC 4493/2013) and all patients signed a written informed consent. The trial was registered at Australian New Zealand Clinical Trial Registry (Trial Id: ACTRN12615000084583).

### Capsule manufacture and coding

Capsules were manufactured by an external compounding pharmacy (Pharmacy 777, Applecross, Perth, WA). They were identical in shape, size and colour and contained leucine- 3 g/d or placebo (lactose - 2.67 g/d). All capsules were then coded and allocated by an investigator who was not involved in either data collection or in analysis (JS). Codes were unscrambled at the end of trial prior to data analysis. Participants randomised to each treatment arm, were required to consume 6 capsules each day; two each with breakfast, lunch and dinner. The participants recorded their capsule consumption daily and noted any missed doses.

### Study diets

Resting metabolic rates (RMR) were measured in each participant via indirect calorimetry (Deltatrac II Metabolic Monitor; Datex-Ohmeda, Instrumentarium Corp, Helsinki, Finland). Energy requirements were calculated for all participants based on 75% of measured RMR multiplied by an activity factor 1.5 for men and 1.3 for women [32] As participants were requested to avoid vigorous exercise during the trial, these PALs would be equivalent to sedentary activity. Biweekly meals plan that included six meals each day (3 main meals+ 3 snacks) were provided to each person and ranged from 1000 to 2800 kcal/day and followed NHMRC guidelines for the target age group for all nutrients (Supplementary S1). We however restricted dairy to 1 serve per day, meat no more than 3 serves per week for men and 2 serves per week for women. This was necessary to restrict total dietary leucine intake to 3 g/d. Calorie-calculated recipes with standard measurements of ingredients were provided to each participant for consistency, and to assist in controlling portion sizes.

### Anthropometric measurements and blood chemistry

Measurements were recorded for waist circumference using a steel tape around mid-point of lowest point of last rib and upper part of pelvic bone. Body composition using DEXA (Prodigy™, Lunar Corporation USA) was measured twice, at the start and end of trial and used for total weight and reporting regional adiposity measurements. Body weight on a calibrated scale and multi frequency Bioimpedence (BIA) Analysis (In Body 3.0, Biospace, Seoul, South Korea) was used each fortnight to track compliance to energy restriction during the trial. Fasting and 2 h (OGTT- 75 g glucose) postprandial venous blood was collected by a trained phlebotomist at the start of the experiment and at the end of 8 weeks. Centrifuged and aliquots of blood was stored at −80 ^ο^C, before being sent to a hospital laboratory for analysis using validated ELISA techniques.

### Compliance

Compliance to the trial was decided on either > 85% capsule ingestion and/or between 1 and 5% weight loss at the end of each month of the 8-weeks trial week. Additionally, all participants were asked to fill in a short survey describing the frequency and serve size of leucine rich foods such as dairy, grain foods, dairy food and nuts/seeds both before and after the trial. The latter information was used to assess their compliance to the prescribed diet. Exercise was not part of our trial, but we requested all our participants to complete a IPAQ (short version) [33] before and after the trial to ensure there was no change in levels of physical activity during the trial. We used BIA machine to measure body composition at 0 week, 3rd week, 5th week and 8th week for compliance. Any participant who did not lose weight or increased weight by the 3rd week was not included in the trial. Those who were stable or exhibited no decrease in body weight during mid trial (5th week) were checked for diet and capsule compliance. They were also booked for a counselling session with trained dietitian (KP). Moreover, fortnightly calls were made to all participants to reassure compliance and identify any additional support required.

### Intervention protocol

The study was conducted by one investigator (KP), and to allow easier project management, the selected participants were split into two groups by studying them in two phases. Phase 1 [*n* = 18: 9 in placebo and 9 in the intervention group] was completed at the end of one year well before the Christmas break, and Phase 2 [*n* = 19] was started well after the following New Year holidays. Participants meeting inclusion criteria and willing to participate were invited for an orientation meeting where they were educated about the study requirements, the tests/measurements that needed to be performed, the equipment to be used, capsule consumptions and meal plans to be followed. A date for baseline measurements and commencement of capsules intake was decided.

Prior to their first measurement day, all participants were asked to consume a dinner which conformed to the trial meal plan provided to them. Participants attended the research centre following at least 10 h of overnight fasting, 8 h of sleep and abstinence from alcohol and vigorous exercise for at least 36 h prior to the measurement. They were instructed not to shower in the morning to avoid any increase in metabolic rate. On arrival, they emptied their bladder and changed into a standard dressing gown, after which their weight and waist circumference were measured. They were then asked to rest in bed for 30 min in the supine position, in a 25 ^ο^C maintained insulated chamber. RMR was measured twice for 25 min each with a 10 min rest in between. We used the second measurement for our analysis. Fasting bloods were then drawn by trained phlebotomists for blood chemistry followed by ingestion of standard glucose drink (75 g). At the conclusion of two hours, blood was drawn again for postprandial measurements. Body composition using DEXA and BIA machines was measured towards the end. All participants were offered beverages and light refreshments before they left the premises. All the participants also completed diet records and physical activity questionnaires on both onsite measurement days (at start and after 8 weeks) in addition to other measurements and capsules intake chart. This was to assure their compliance to diet and physical activity guidelines was maintained. Capsules sufficient for four weeks were provided on the first measurement day and refilled during their fortnightly visits. Additional dietary counselling was provided if poor compliance was identified during fortnightly visits, (either > 85% capsule ingestion and/or between 1 and 5% weight loss). Additional phone calls were made to check diet and capsule compliance throughout the trial. All participants returned their capsule containers at the end of 8 weeks. Any missed doses were counted and recorded.

## Statistical methods

### Sample size calculations

Sample size was calculated utilising GPower version 3.1.9.2 [34]. Based on 2 × 2 repeated measures, to detect a small effect of 0.25 with a power of 80% at the 5% significance level and 0.5 correlation between measures, the total number of participants was estimated as 34 (17 for each group).

### Analysis

All analyses were performed by using IBM SPSS Statistics for Windows (version 26 Armonk, NY: IBM Corp). Normality was assessed and a natural logarithm transformation was applied for skewed variables. To investigate the effect of leucine supplementation (Treatment) at 8 weeks on body composition and fasting metabolic parameters, we performed an analysis of covariance (ANCOVA) (via General Linear Model (GLM) univariate procedure in SPSS), using the outcome variables at 8 weeks as the dependent variable, with an adjustment of the baseline values. In our analyses, age, gender, phase and energy deficit, kJ/d were adjusted as confounders and two potential interaction effects between treatment and gender (treatment*gender), and between treatment and phase (1 & 2) (treatment*phase) were assessed in the ANCOVA. The study was conducted in 2 phases (spring-summer (Phase 1) and Autumn-winter (Phase 2) for feasibility of clinical trial, which differed in season, gender distribution and we were interested in knowing if that has any potential difference to the outcome. As normality could not be achieved even after transformation for several outcome variables, bootstrapping method with 1000 bootstrap samples was used for deriving robust estimates of standard errors, regression coefficients and corresponding 95% confidence intervals. A *p*-value of less than 0.05 was accepted as being statistically significant at 5% level. In addition, a “Change” variable of each main outcome variable between the values at baseline and at 8 weeks was calculated and used in multivariable linear regression analysis for verifying the findings achieved via the ANCOVA (data not shown). Both analyses revealed similar direction and magnitude of the intervention effect, and hence only those based on the ANCOVA were reported in the paper.

## Results

**Fig. 1:**
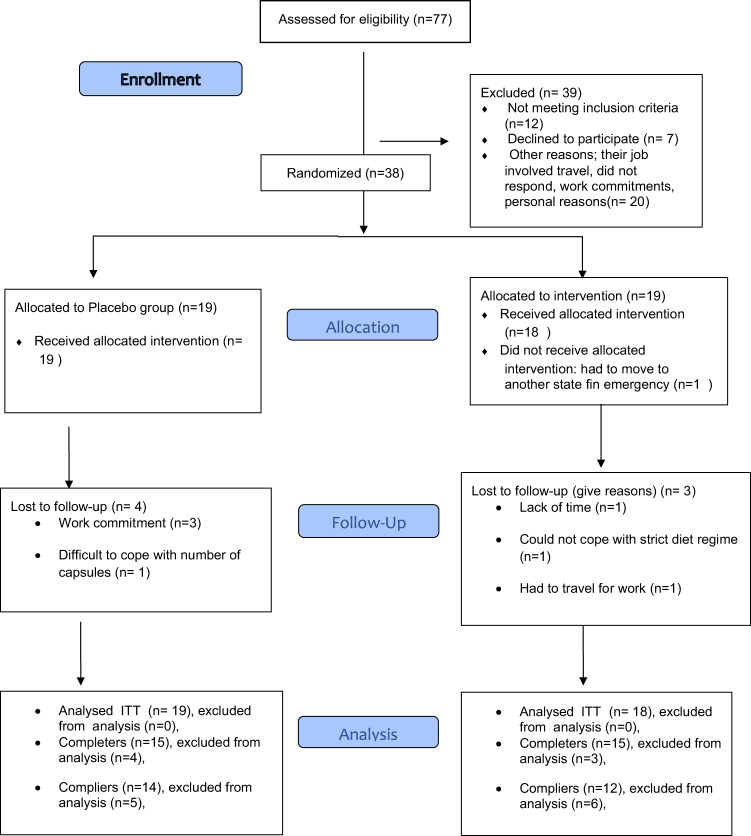
CONSORT Flow Diagram. Participant enrolment, allocation, follow-up and analysis.

Out of 77 interested responder, 38 passed eligibility criteria for this study and were recruited and randomised for allocation to the experimental groups. However, one participant withdrew before start of the study due to work commitments. Fifteen participants from each group completed the study. Three participants in the intervention group and 4 participants from the placebo group were lost to follow-up due to either work commitments or inability to adhere to the dietary regime. In this study, ITT methodology was used to include any participants missing final measurements but attended the trial for 4 weeks or more (Fig. 1). The placebo group included 12 females (63.2%) and 7 males (36.8%) and the intervention group consisted of 13 females (72.2%) and 5 males (27.8%). Baseline characteristics for the 37 total participants are presented in Table 1 and both groups were similar in all characteristics and metabolic measures.

**Table 1:** Baseline anthropometric and metabolic characteristics of the participants.

Variables	Placebo (*n* = 19)	Leucine (*n* = 18)	*P*-value^a^
Age (yrs)	46.5 ± 14.59	51.3 ± 14.79	0.326
Gender (M/F)	7 M/12 F	5 M/13 F	0.556^b^
Weight (kg)	92.5 ± 19.42	94.0 ± 21.43	0.826
Fat mass (kg)	38.1 ± 11.55	41.8 ± 11.76	0.349
Fat free mass (kg)	54.4 ± 13.00	52.2 ± 13.86	0.63
Fat %	41.3 ± 9.89	45.1 ± 6.96	0.191
Lean tissue mass (kg)	51.5 ± 12.55	49.5 ± 13.41	0.634
Appendicular Mass (kg)	23.2 ± 5.51	21.0 ± 5.10	0.204
Non- appendicular mass (kg)	28.3 ± 7.22	28.5 ± 9.49	0.939
Waist circumference (cm)	103.0 ± 14.75	106.0 ± 15.57	0.546
Stumvoll ISI (fasting)	14.0 ± 6.14	16.0 ± 7.82	0.402
Fasting Glucose (mmol/l)	5.5 ± 0.63	5.5 ± 0.97	0.765
Fasting Insulin (mU/L)	9.57 ± 6.302	8.13 ± 4.332	0.426
Number of MetS components	2.4 ± 1.07	2.9 ± 1.31	0.149
Prescribed Calorie Deficit (kJ/d)	−2387 ± 616.1	−2336 ± 612.1	0.940

All values are mean ± SD. *ISI* Insulin sensitivity index, *SD* Standard deviation.

^a^*P*-value: Independent samples t-test.

^b^Chi Square test for categorical variables.

**Fig. 2:**
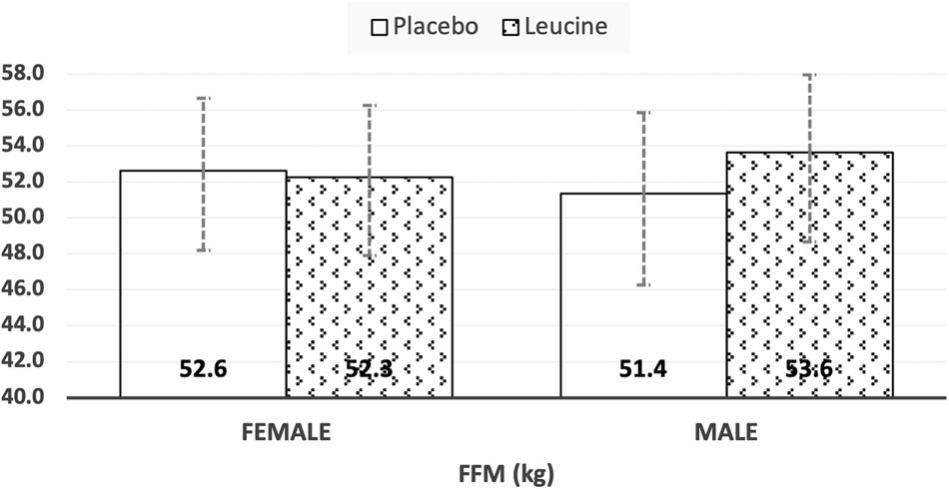
Estimated marginal means (with 95% CI) of adjusted fat free mass (FFM) following leucine supplementation during energy restriction.

**Fig. 3:**
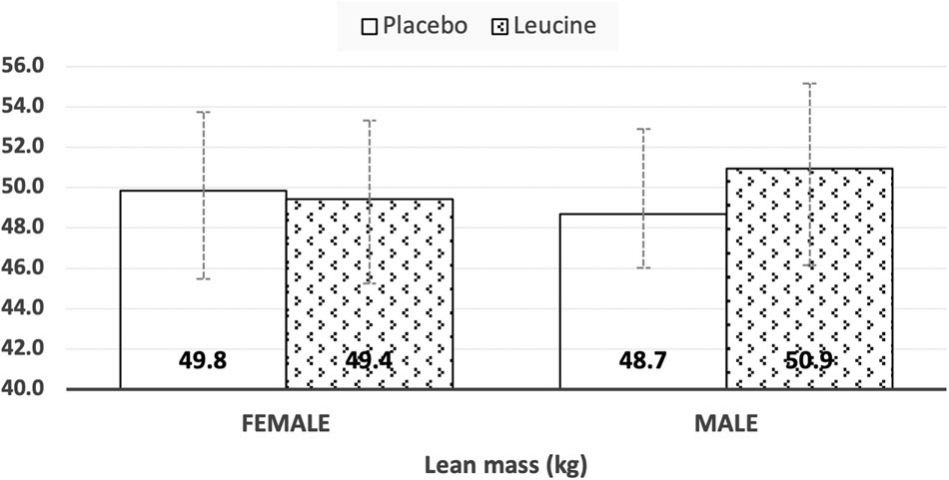
Estimated marginal means (with 95% CI) of adjusted lean tissue mass (LTM) following leucine supplementation during energy restriction.

In this paper we report the results based on the ANCOVA, in which the effect of treatment on the outcome variables post supplementation (at the end of 8 weeks) was assessed by adjusting for respective baseline (starting) values and other confounders. Weight loss among Leu (94.00 ± 21.43 vs 90.60 ± 22.32 kg) and placebo (92.50 ± 19.42 vs 88.21 ± 20.33 kg) groups were similar at the end of intervention period. For body composition parameters, there was a significant between-group difference in FFM following 8 weeks of weight loss (placebo: 51.99 ± 2.19 kg vs Leu: 52.95 ± 2.13 kg, *p* = 0.045) and LTM trended towards significance (placebo: 49.24 ± 2.10 kg vs Leu: 50.17 ± 2.05 kg, *p* = 0.050) (Table 2). There was a treatment x sex interaction for both variables (FFM: *p* = 0.040; LTM: *p* = 0.045), and the estimated marginal means showed that compared to their placebo counterparts, males of the Leu group had a higher FFM and LTM while females had similar FFM and LTM (Figs. 2 and 3). This shift in LTM composition was significant in the non-appendicular tissue mass component compared to appendicular tissue mass (Table 2).

**Table 2:** Effects of Leucine supplementation on anthropometry and body composition following 8 weeks of an energy restricted diet.

	Placebo (*n* = 19)		Leucine (*n* = 18)		ANCOVA^#^				
Variable									
(Measured at end)	Mean (std. error)		Mean (std. error)		Treatment effect	Sex effect	Treatment x Sex interaction	Phase effect	Treatment x Phase interaction
		95% CI		95% CI					
Weight (kg)	88.79 (3.66)	(81.66, 96.24)	89.47 (3.63)	(82.49, 97.26)	NS	NS	NS	NS	NS
WC (cm)	99.55 (2.96)	(93.85,105.56)	99.43 (2.70)	(94.06,105.03)	NS	NS	NS	*P* = 0.021	NS
% Fat mass	40.37 (1.51)	(37.49, 43.25)	41.42 (1.41)	(38.86, 44.24)	NS	*P* = 0.030	NS	NS	NS
Fat mass (kg)	36.49 (2.26)	(32.33, 41.15)	36.69 (2.10)	(32.46, 41.02)	NS	NS	NS	NS	NS
Fat free mass (kg)	51.99 (2.19)	(47.85, 56.70)	52.95 (2.13)	(49.05, 57.40)	*P* = 0.045	NS	*P* = 0.040	NS	NS
Lean tissue mass (kg)	49.24 (2.10)	(45.29, 53.72)	50.17 (2.05)	(46.43, 54.45)	*P* = 0.050	NS	*P* = 0.045	NS	NS
Appendicular mass (kg)	22.30 (0.84)	(20.62, 24.02)	22.42 (0.76)	(20.91, 23.93)	NS	NS	NS	NS	NS
Non-Appendicular mass (kg)	27.42 (1.38)	(25.05, 30.57)	27.63 (1.46)	(24.92, 30.74)	NS	NS	*P* = 0.023	NS	NS

*WC* Waist circumference, *std. error* Standard error, *NS* Statistically non-significant statistically (*P* > 0.05).

Bootstrapping method with 1000 bootstrap samples was used for deriving *p*-values, parameter estimates, standard errors and 95% confidence intervals for variables which normality was not assumed.

^#^Variables included after adjusting for baseline values: age, gender, treatment, phase and energy deficit, treatment*sex, and treatment*phase (an interaction term was removed if it was non-significant).

Mean for Placebo and Leucine were estimated based on the ANCOVA model by substituting the mean values of age and energy deficit, and corresponding baseline mean values.

There was no marked improvement in insulin sensitivity (fasting and postprandial glucose and insulin, McAuleys ISI, Stumvoll fasting and postprandial), and MetS component post supplementation (Table 3). However, we observed a significant difference in postprandial insulin (*p* = 0.025) and Stumvoll index (*p* = 0.041) between the phases, respectively. There was also a significance interaction noticed between treatment groups and phases for MetS component (*p* = 0.037).

**Table 3:** Effect of Leucine supplementation on metabolic parameters following 8 weeks of energy restriction.

Variables	Placebo (*n* = 19)		Leucine (*n* = 18)		ANCOVA^#^				
(Measured at end)	Mean (std. error)		Mean (std. error)		Treatment effect	Sex effect	Treatment x Sex interaction	Phase effect	Treatment x Phase interaction
		95% CI		95% CI					
Fasting glucose (mmol/L)	5.2 (0.11)	(5.06, 5.48)	5.5 (0.15)	(5.22, 5.76)	NS	NS	NS	NS	NS
Post prandial glucose (mmol/L)	6.6 (0.33)	(5.95, 7.24)	6.8 (0.40)	(6.06, 7.61)	NS	NS	NS	NS	NS
Fasting insulin (mU/L)	8.19 (1.12)	(6.19, 10.66)	8.26 (0.10)	(6.46, 10.42)	NS	NS	NS	NS	NS
2 h PP insulin(mU/L)	39.07 (5.65)	(28.82, 50.99)	42.77 (6.24)	(31.26, 55.73)	NS	NS	NS	*P* = 0.025	NS
McAuleys	8.12 (0.44)	(7.24, 8.96)	7.86 (0.43)	(7.03, 8.75)	NS	NS	NS	NS	NS
ISI, Stumvoll F	20.79 (3.31)	(15.16, 28.29)	18.16 (2.64)	(13.75, 24.16)	NS	NS	NS	NS	NS
ISI Stumvoll PP	3.21 (0.54)	(2.20, 4.32)	3.69 (0.62)	(2.52, 5.00)	NS	NS	NS	*P* = 0.041	NS
MetS component	2.3 (0.16)	(1.98, 2.64)	2.2 (0.18)	(1.86, 2.59)	NS	NS	NS	NS	*P* = 0.037

*F* Fasting, *PP* Postprandial, *NS* Statistically non-significant (*P* > 0.05).

Bootstrapping method with 1000 bootstrap samples was used for deriving *p*-values, parameter estimates, standard errors and 95% confidence intervals for variables which normality was not assumed.

^#^Variables included after adjusting for baseline values: age, sex, treatment, phase, change in FM and change in FFM, change in WC, treatment*sex, and treatment*phase (an interaction term was removed if it was non-significant).

## Discussion

A small decrease in weight (5–10%) can bring about significant reductions in TAGs, blood pressure, fasting blood glucose and insulin, HcA1C, and increments in HDL-C [35, 36]. There is a close relationship of excessive adiposity to insulin resistance and hence MetS. It follows that fat loss per se could elicit significant improvements in insulin sensitivity [37]. Preserving FFM, specifically, lean mass during intentional weight loss is important [38], as it also impinges on insulin sensitivity. Supplementation with BCAA during energy restriction, particularly leucine, may serve the purpose of LTM retention [20, 30]. We are unaware of any published study that has addressed these issues in MetS, while exploring the changes in body composition and insulin sensitivity markers following leucine supplementation.

A systematic review examined the loss in FFM with various weight loss strategies, such as energy restriction, exercise, or surgery [31]. Linear regression analysis revealed very low-calorie diets resulted in significant loss in %FFM compared to exercise and surgical interventions. The loss was greater among men (27 ± 7%) than women (20 ± 8%, *P* = 0.08) [8]. A more recent study provided the range of expected loss of FFM to total decrease in weight as 35–40% for men and 30–35 % for women [39].

The present study demonstrates that although both groups lost weight at the end of the intervention period, leucine supplementation dampened the drop in FFM and LTM associated with an energy-restricted diet (Table 2). This is consistent with a recently published RCT that employed a much higher dose (10 g/d) of leucine [40]. We however make a novel observation of a treatment x sex interaction in our outcomes (Table 2). This interaction indicated that men retained more FFM and LTM following weight loss as compared to women (Figs. 2 & 3). This sex effect of Leu treatment was more in the non-appendicular tissue mass compartment, rather than appendicular tissue mass. The latter compartment makes up ~80% of total skeletal muscle mass and would be the expected site to observe leucine effects. The non-appendicular compartment is comprised of all the organ tissue masses (liver, kidney, spleen etc) and truncal skeletal muscle mass. Leucine acts on a variety of tissues in the body and while it is expected to stimulate protein synthesis in skeletal muscle, in organ tissues like the liver, it serves instead to dampen protein degradation. Under basal, weight stable conditions gender differences in protein turnover have been reported in individual studies, but a review of evidence negates the presence of a gender bias [35]. In effect, those authors concluded that better control over subject selection, methodology employed (phenylalanine versus leucine trace), and accounting for relative body fatness in the analysis was necessary to confirm a gender bias in protein turnover [35]. It is difficult to explain the observations of preserved LTM only in men. The much smaller number of men versus women studied could make this a chance finding.

Other studies suggest that the extent of loss of FFM and FM with leucine supplementation may be dependent on many factors such as age, gender, health status of individuals, dosage and length of supplementation and presence of energy restriction with and without exercise [8, 40, 41]. One study on mice and another on elderly men and women with rheumatoid arthritis both suggest that gain in fat mass can be blunted with leucine supplementation [42]. However, due to either small sample size, dose, variation in age of subjects or duration we did not notice any difference in FM between the groups (Table 3).

### Leucine and insulin sensitivity

FFM is metabolically active tissue and has been linked with insulin-stimulated glucose uptake in central and peripheral tissues [43]. Circulating insulin has the capacity to enhance protein synthesis [44]. Some studies show positive association between FFM and insulin sensitivity in older adults [45, 46] and following calorie-restricted weight loss [47]. Overall, whilst there are animal studies to suggest leucine supplementation may contribute to improved insulin sensitivity [25], evidence in humans is lacking [41]. In this study we did not observe any improvement in glucose tolerance or surrogate markers of insulin sensitivity after controlling for changes in body composition (Table 3). There is the possibility of multiple mechanisms being involved in leucine’s improvement of glucose metabolism. One of them is the potential of a combined action of leucine and its metabolites - α-ketoisocaproate (α-KIC) and β-hydroxy-β-methylbutyrate (HMB)- that are formed in skeletal muscle, on increasing protein synthesis and regulating glucose homeostasis Interestingly, it has also been observed that improved BCAA intake augments levels of plasma BCAA which are inversely related to insulin sensitivity [48]. On the other hand, weight loss per se increases insulin sensitivity that reduces proteolysis and thereby decreases plasma BCAA [49]. Hence, the debate of improved insulin sensitivity and high protein/BCAA diets remains equivocal, and further investigation is required.

### Strengths & limitations

The strengths of this investigation are the excellent trial design and the double blinding of both participants and investigators. The testing of compliance to all aspects of intervention every fortnight, an individualized weight loss strategy, provision of sample menus and telephone contact to assist adherence to study requirements, were other favourable facets. We acknowledge that the short duration of the trial may not have allowed the full impact of leucine on all aspects of body composition, as well as changes in insulin sensitivity markers. Additionally, the gender bias observed needs replication as fewer men than women were studied.

## Conclusions

Leucine supplementation during weight loss prevented the loss of FFM and LTM in those at risk of MetS, but this effect was only significant in men. There was no further effect on glucose tolerance or insulin sensitivity on accounting for the changes in body composition following energy restriction.

### Supplementary information

Table S1.

## Data Availability

The raw data used in this paper is freely available for non-commercial purposes only. The institution’s human ethics committee must endorse the formal request, and researchers may contact Mario Soares m.soares@curtin.edu.au in the first instance.
