# Supplementary material for: The impact of leucine supplementation on body composition and glucose tolerance following energy restriction: an 8-week RCT in adults at risk of the metabolic syndrome
Source: Eur J Clin Nutr. 2023 Nov 3;78(2):155–62. doi: 10.1038/s41430-023-01360-1 (PMC10853066; doi:10.1038/s41430-023-01360-1)
Supplement: Supplementary file 1 — Table S1. [file 41430_2023_1360_MOESM1_ESM.docx]

S1- Two-day Sample Meal Plan – Leucine Supplementation Study

|  | Day One | Day Two |
| --- | --- | --- |
| Breakfast | 1 egg w mushrooms and tomato  Black tea/coffee | Oats (50 g) w milk (50 ml) & cinnamon sprinkle  Black tea/coffee |
| Snack | Yoghurt (100 g) + 1 Fruit | 1 c of fruit salad |
| Lunch | Grilled chicken (60g) + rice (50g) & vegetables of your choice | Lean steak sandwich w vegetables (60g see recipe) |
| Snack | 6-8 pcs mixed nuts | 1 fruit + yoghurt (100 g) |
| Dinner | Lentil & potato (see recipe) patties + stir fry vegetables | Spinach & lentils (see recipe) soup+ roast potato ( 1 medium) |
| Snack | 1 c of fruit salad | Diet jelly |
